# Terry’s Nails Without Systemic Disease: A Case Report of a Unique Clinical Sign

**DOI:** 10.7759/cureus.88172

**Published:** 2025-07-17

**Authors:** Abdulrahman Saleh Aldairi, Faris Alsaedi, Deema Hani Beheiry, Mohammad Hakim, Ibrahim Allihibi, Homaid Alotaibi

**Affiliations:** 1 Department of Dermatology, King Faisal Hospital, Ministry of Health, Makkah, SAU; 2 Faculty of Medicine, Umm Al-Qura University, Makkah, SAU

**Keywords:** apparent leukonychia, fingernails and toenails, idiopathic leukonychia, idiopathic terry’s nails, isolated nail abnormality, nail manifestation without comorbidity, non-systemic terry’s nails, terry's nails

## Abstract

Terry’s nails are a clinical sign of apparent leukonychia, marked by proximal nail whitening and a preserved distal band, commonly linked to systemic diseases such as liver cirrhosis and chronic kidney disease. We report a case of a 26-year-old man with asymptomatic leukonychia of all fingernails and toenails since early childhood. Physical examination revealed features consistent with Terry’s nails. A comprehensive systemic evaluation was unremarkable. This case demonstrates that idiopathic Terry’s nails, though extremely rare, can occur in otherwise healthy individuals.

## Introduction

Terry’s nails represent a form of apparent leukonychia, characterized by a pale, ground-glass appearance affecting most of the nail plate. The lunula is typically absent, with only a narrow distal band of normal pink coloration remaining [1]. This clinical sign was first described by Dr. Richard Terry in 1954 in patients with liver cirrhosis [2]. Subsequent studies have associated Terry’s nails with systemic conditions such as congestive heart failure, chronic kidney disease, and diabetes mellitus [1]. Although the exact pathophysiology is unknown, it is thought to include vascular abnormalities, as well as modifications to the nail bed's connective tissue [3-5]. However, the presence of Terry’s nails in otherwise healthy individuals with no identifiable systemic disease is exceedingly rare [3]. In this report, we present a case of idiopathic Terry’s nails involving both fingernails and toenails in a patient with no clinical or laboratory evidence of underlying illness.

## Case presentation

**Figure 1:**
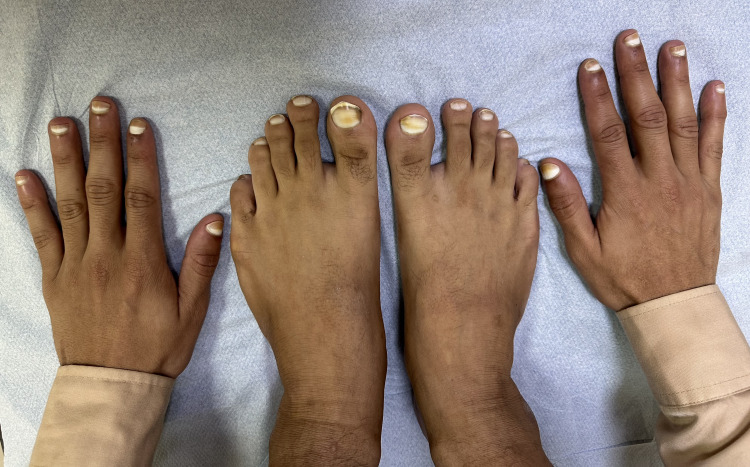
Clinical image of Terry’s nails showing bilateral involvement of all fingernails and toenails. The patient's nails exhibit a characteristic ground-glass whitening of the proximal nail plate involving approximately 80% of the nail surface, with preservation of a narrow, well-demarcated distal pink-to-brownish band. Lunulae are not visible.

A 26-year-old man was referred to the dermatology clinic by his primary care physician due to a long-standing history of asymptomatic whitish discoloration affecting both fingernails and toenails, first noticed during early childhood. This was his first dermatology consultation, and he had not undergone prior medical or dermatological evaluation for this condition. A comprehensive medical history was obtained to assess for possible underlying systemic illnesses, including cardiovascular, hepatic, hematological, and metabolic diseases, such as diabetes mellitus. The patient denied alcohol consumption and reported no family history of similar nail abnormalities. Physical examination revealed apparent leukonychia involving approximately 80% of the proximal nail plate in all fingernails and toenails, with preservation of a narrow distal pink to brownish band. The lunulae were not visible. The white discoloration did not change with pressure, consistent with apparent leukonychia (Figure 1).

A provisional diagnosis of Terry’s nails was made based on the clinical appearance. A thorough systemic evaluation was conducted to exclude known comorbidities associated with Terry’s nails. Laboratory investigations, including complete blood count with differential, liver and renal function tests, hepatitis B serology, hepatitis C antibody, HIV p24 antigen test, hemoglobin A1c, erythrocyte sedimentation rate (ESR), and C-reactive protein (CRP), were all within normal limits. Imaging studies, including chest radiography and transthoracic echocardiography, revealed no abnormalities. Histopathological examination was not performed, due to the classical presentation and absence of clinical suspicion for alternative diagnoses. The patient was counseled regarding the benign nature of the condition and reassured. No specific treatment was required. Based on the characteristic clinical findings and exclusion of underlying systemic causes, a diagnosis of idiopathic Terry’s nails was made.

## Discussion

This case describes an instance of idiopathic Terry’s nails in a healthy individual with no clinical or laboratory evidence of systemic disease. Terry’s nails are commonly described in the literature as a nail abnormality associated with a variety of systemic illnesses, including liver cirrhosis, congestive heart failure, chronic kidney disease, diabetes mellitus, Reiter’s syndrome, and pulmonary tuberculosis [4-6]. Agrawal and Beniwal reported the only previous case of idiopathic Terry’s nails, which was limited to the fingernails [3]. To the best of our knowledge, this is the first documented case involving both fingernails and toenails in an otherwise healthy individual, thus expanding the clinical spectrum of this sign.

The classic presentation of Terry’s nails involves the absence of the lunula and diffuse proximal nail whitening that abruptly terminates 1-2 mm from the distal edge, forming a narrow red to brown band, typically corresponding to the onychodermal junction [4]. This pattern generally appears symmetrically on both hands [5].

The underlying pathogenesis of Terry’s nails remains incompletely understood, though both vascular and connective tissue alterations have been proposed. It is hypothesized that reduced vascularity in the proximal nail bed, possibly due to capillary bed compression by fibrous tissue or altered dermal collagen, leads to the pale appearance [5,6]. Histopathologic studies have demonstrated reduced capillary density and prominent distal telangiectasia [4].

A study by Fernandez-Somoza et al., using capillaroscopy in patients with liver disease and proximal apparent leukonychia, found distinctive capillary loop abnormalities and loss of vascular definition, further supporting vascular pathophysiology even in variants of Terry’s nails [7]. While our patient’s idiopathic presentation lacked clinical justification for biopsy or dermoscopy, these tools may serve as non-invasive or histologic adjuncts in diagnostically uncertain cases. In our case, the decision not to pursue biopsy was based on the characteristic appearance, absence of systemic findings, and benign clinical course.

Differentiating Terry’s nails from other forms of leukonychia is essential. Lindsay’s nails (half-and-half nails) present with a proximal white portion and a sharply demarcated distal brown band, involving approximately 20%-60% of the nail. Muehrcke’s lines appear as paired transverse white bands across the nail that do not move with nail growth and are often associated with hypoalbuminemia or chemotherapy exposure. In contrast, true leukonychia (as opposed to apparent) involves white discoloration within the nail plate itself and typically moves distally as the nail grows [1,5]. Table 1 provides a visual summary comparing these nail findings.

**Table 1:** Comparison of Terry’s nails, Lindsay’s nails, and Muehrcke’s lines. CHF, Congestive Heart Failure; CKD, Chronic Kidney Disease; DM, Diabetes Mellitus

Feature	Terry’s Nails	Lindsay’s Nails (Half-and-Half)	Muehrcke’s Lines
Appearance	Diffuse proximal whitening with narrow distal band	Proximal white area with distal brown-red band	Paired transverse white lines
Lunula	Absent	Present or absent	Present between bands
Etiology	Apparent leukonychia; vascular/connective tissue	Chronic kidney disease	Hypoalbuminemia, chemotherapy
Association With Disease	Liver disease, CHF, CKD, DM, others	Chronic renal failure	Nephrotic syndrome
Movement With Nail Growth	No	No	No
Histopathology	Distal telangiectasia, reduced vascularity	Variable	Normal nail plate, subungual changes

## Conclusions

To the best of our knowledge, this is the first documented case in the literature of Terry’s nails involving both fingernails and toenails in an otherwise healthy individual without underlying systemic disease. This case expands the known clinical spectrum of Terry’s nails and reinforces that, although uncommon, idiopathic variants do exist. It underscores the importance of conducting a comprehensive systemic evaluation in patients presenting with nail findings suggestive of Terry’s nails to exclude potential underlying comorbidities. Further documentation and investigation of similar idiopathic presentations may enhance our understanding of their pathophysiology and clinical significance.

## References

[1] Lin CP, Alkul M, Truitt JM, Stetson CL (2020). Development of Terry's nails after a gastrointestinal bleed. Proc (Bayl Univ Med Cent).

[2] Meegada S, Verma R (2020). Terry's nails. Clin Case Rep.

[3] Agrawal A, Beniwal R (2024). Terry's nails. Indian J Dermatol Venereol Leprol.

[4] Nia AM, Ederer S, Dahlem KM, Gassanov N, Er F (2011). Terry's nails: a window to systemic diseases. Am J Med.

[5] Witkowska AB, Jasterzbski TJ, Schwartz RA (2017). Terry’s nails: a sign of systemic disease. Indian J Dermatol.

[6] Pitukweerakul S, Pilla S (2016). Terry’s nails and Lindsay’s nails: two nail abnormalities in chronic systemic diseases. J Gen Intern Med.

[7] Fernandez-Somoza JM, Ginarte M, Otero E, Tomé S, Soutullo C, Martínez-Ulloa A, Gonzalez-Quintela A (2021). Clinical and capillaroscopic findings in patients with liver disease and proximal apparent leukonychia (Terry nails and its variants). Medicine (Baltimore).

